# Influence of Geocomposite Properties on the Crack Propagation and Interlayer Bonding of Asphalt Pavements

**DOI:** 10.3390/ma14185310

**Published:** 2021-09-15

**Authors:** Sara Spadoni, Lorenzo Paolo Ingrassia, Giulio Paoloni, Amedeo Virgili, Francesco Canestrari

**Affiliations:** 1 Department of Civil and Building Engineering and Architecture (DICEA), Università Politecnica delle Marche, Via Brecce Bianche, 60131 Ancona, Italy; s.spadoni@pm.univpm.it (S.S.); a.virgili@univpm.it (A.V.); f.canestrari@univpm.it (F.C.); 2 Autostrade per l’Italia, Via A. Bergamini 50, 00159 Roma, Italy; gpaoloni@autostrade.it

**Keywords:** geocomposite, reinforced asphalt pavement, reflective cracking, maintenance and rehabilitation, Falling Weight Deflectometer (FWD), full-scale trial section

## Abstract

The application of geocomposites as reinforcement in asphalt pavements is a promising solution for the maintenance/rehabilitation of existing pavements and for the construction of new pavements, whose effectiveness strongly depends on the physical and mechanical properties of the geocomposite. This study aims at assessing the influence of four different geocomposites, obtained by combining a reinforcing geosynthetic with a bituminous membrane, on the crack propagation and interlayer bonding of asphalt pavements. First, a laboratory investigation was carried out on double-layered asphalt specimens. The crack propagation resistance under static and dynamic loads was investigated through three-point bending tests (carried out on specimens with and without notch) and reflective cracking tests respectively, whereas the interlayer shear strength was evaluated through Leutner tests. Then, a trial section was constructed along an Italian motorway and a Falling Weight Deflectometer (FWD) testing campaign was carried out. The laboratory investigation highlighted that—as compared to the unreinforced system—the geocomposites increased the crack propagation energy in the layer above the reinforcement from five to ten times, indicating that they can significantly extend the service life of the pavement by delaying bottom-up and reflective cracking. However, they also worsened the interlayer bonding between the asphalt layers (de-bonding effect). The field investigation indicated that all geocomposites decreased the stiffness of the asphalt layers with respect to the unreinforced pavement as a consequence of the de-bonding effect, thus corroborating the laboratory results. Based on the results obtained, it is desirable that the geocomposite possess a high energy dissipation capability and an upper coating ensuring good adhesion between the asphalt layers. The monitoring of the existing trial section in the future will provide useful data on the long-term field performance of reinforced pavements subjected to actual motorway traffic.

## 1. Introduction

Geosynthetics have been in use since the 1970s and in the first applications they were applied between base and subgrade materials with functions of separation, filtration and drainage. Nowadays, a large variety of geosynthetics are available on the market, each one designed to fulfil one or more specific functions. In recent years, geosynthetics with a reinforcement usually made of polymeric, steel or glass grids have been developed, and the benefits deriving from their use are increasingly gaining attention, both for the maintenance/rehabilitation of existing pavements and the construction of new pavements [1].

Through laboratory investigations, field investigations as well as numerical simulations, many researchers have demonstrated that geosynthetics can improve the overall pavement performance. The main benefit provided by geosynthetics is the delay of crack propagation, both in the case of new pavements (bottom-up cracking) and in the case of existing cracked pavements (reflective cracking), which results in a significant increase of the service life and/or in a delay of the necessity of maintenance/rehabilitation operations [2,3,4,5,6,7,8]. At the same time, reinforcements can improve the rutting performance as compared to the unreinforced pavement [9,10,11]. The use of sensors to monitor the structural response of reinforced pavements indicates that such improvements are ascribable to the mitigation of the strain level within the pavement [10,12,13]. In addition, the application of geosynthetics for pavement maintenance/rehabilitation represents an economic and sustainable choice because it can allow the replacement of lower thickness of asphalt layers, thus reducing the amount of asphalt concrete that must be discarded and produced, resulting in lower emissions, reduced intervention time and faster opening to traffic. On the other hand, it should be considered that the reinforcement inevitably represents a discontinuity in the pavement and may cause a reduction of the shear and flexural strength due to a de-bonding effect between the layers. For this reason, the reinforcement is usually installed at a certain depth from the pavement surface, in order to avoid the possible slippage due to the shear stresses induced by traffic loadings (whose value decreases as the depth increases) [6,14,15,16].

Under certain conditions, a promising solution for the maintenance/rehabilitation or the construction of flexible pavements is represented by geocomposites, which are particular types of geosynthetics made of a reinforcement combined with a bituminous membrane. The main advantage of geocomposites is the combination of the reinforcement benefits, i.e., improvement of tensile properties and resistance against the pavement distresses, with the typical advantages of bituminous membranes, which ensure better adhesion between the layers, stress absorbing membrane interlayer (SAMI) effect as well as waterproofing for the underlaying granular layers (i.e., foundation and subgrade). Another important advantage of using geocomposites instead of geosynthetics is that the bituminous membrane protects the reinforcement against the possible deterioration caused by field compaction activities and high working temperatures of asphalt concrete (i.e., 140–170 °C), which may cause the reduction of the mechanical properties of the reinforcement [5]. Furthermore, in the case of maintenance/rehabilitation activities, the application of a geocomposite does not require the construction of a levelling course, unlike traditional geosynthetics. In this sense, geocomposites are particularly advantageous for the activities that are carried out at night to limit the inconvenience to traffic (for instance in the case of motorways and high-speed roads).

However, although many studies have documented the effectiveness of geosynthetics so far, there is still little scientific literature on the use of geocomposites. The results available in literature are encouraging and suggest that geocomposites can effectively delay bottom-up, top-down and reflective cracking, as well as improve the rutting resistance, even though the performance is strongly affected by the interlayer bonding ensured by the geocomposite [17,18,19,20,21,22]. Nevertheless, it is not yet fully understood how the physical and mechanical properties of the geocomposite affect the performance of the reinforced system. Moreover, most research on asphalt systems reinforced with geocomposites has been carried out mainly at the laboratory scale rather than at the field real scale so far, and therefore the laboratory results need to be validated through the construction and monitoring of full-scale trial sections.

Given this background, this paper focuses on the use of geocomposites between asphalt layers. Specifically, the research presented aims at assessing the influence of four geocomposites (with different physical and mechanical properties) on the crack propagation and interlayer bonding of asphalt pavements. The study included a laboratory investigation on double-layered specimens supported by a field investigation on a full-scale trial section. A reference unreinforced system was considered in both cases.

In the laboratory, the crack propagation resistance was investigated by means of static three-point bending tests (simple and fast approach) and dynamic reflective cracking tests (time-consuming approach but more representative of the field conditions thanks to the use of a customized testing configuration). The interlayer bonding was evaluated by means of Leutner tests. In the field, non-destructive Falling Weight Deflectometer (FWD) tests were performed to assess the effect of the geocomposite properties on the stiffness of the asphalt layers.

## 2. Laboratory Investigation

### 2.1. Materials

**Figure 1:**
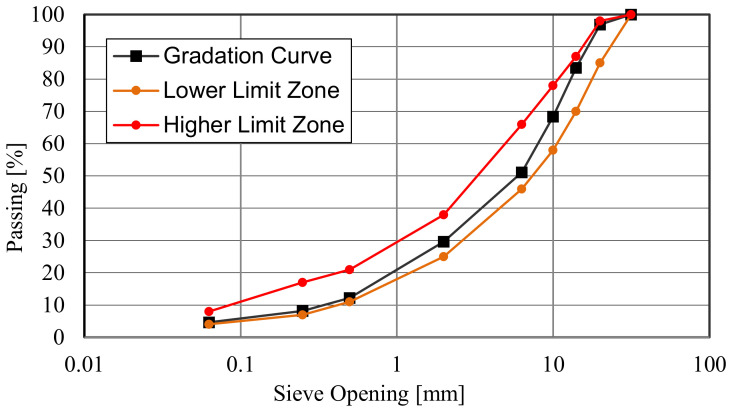
Gradation curve.

The asphalt concrete mix used for the specimen preparation was for binder layers and contained 25% by weight of reclaimed asphalt pavement (RAP). It was characterized by maximum dimension size of 20 mm and bitumen content (styrene-butadiene-styrene (SBS) polymer modified “hard”) of 4.8% by aggregate weight. Its gradation curve is shown in Figure 1.

**Figure 2:**
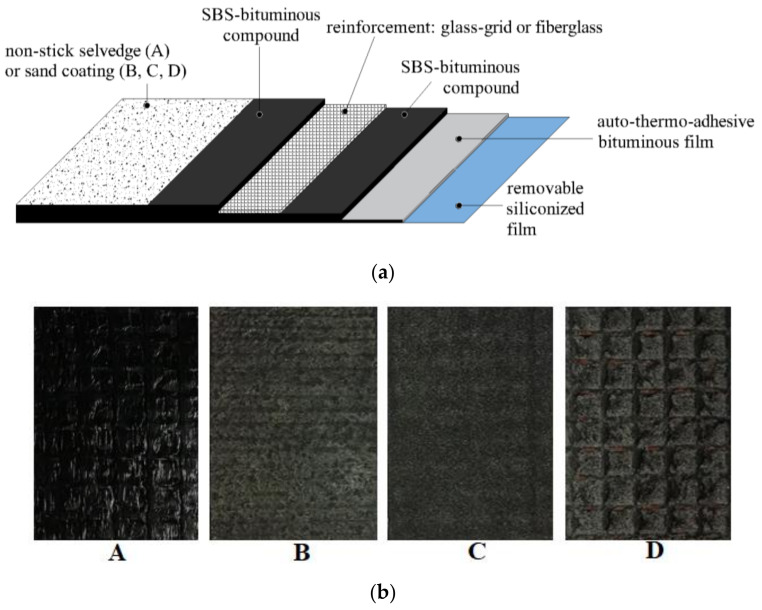
Scheme of the cross-section (**a**) and upper surfaces (**b**) of the geocomposites.

Four geocomposites (hereafter named A, B, C, D), obtained combining a reinforcement with a bituminous compound/membrane, were investigated in this study. A scheme of the cross-section is shown in Figure 2a. All the geocomposites had a nominal thickness of 2.5 mm and they were flexible (unlike grids, which need to be installed flat on the milled surface to work properly). The bituminous compound was manufactured with a SBS polymer modified bitumen. The lower layer was provided with an auto-thermo-adhesive bituminous film protected by a removable siliconized film; in the upper surface of B, C and D, the bituminous compound was coated with fine sand, whereas A had a non-stick selvedge. Figure 2b shows some pictures of the upper surfaces of the four geocomposites. Adhesion was ensured in the first instance by the auto-thermo-adhesive bituminous film and then by the SBS membrane which melted at the compaction temperature; no tack-coat was required. The main difference between the geocomposites was the type of reinforcement: A and D had a glass-grid with a woven-non-woven fabric made of polyester, B had a multi-directional fiberglass and C had an anisotropic glass-grid. The mechanical properties of the geocomposites in the longitudinal (L) and transverse (T) directions according to the product datasheets are given in Table 1.

For the preparation of the unreinforced specimens (coded as N), a tack-coat was necessary to ensure the adhesion at the interface between the two asphalt layers. A cationic emulsion, prepared with traditional bitumen dosed at 55% of the total weight of the emulsion and characterized by low-medium breaking velocity, was used. According to EN 13808 [23], the emulsion employed is classified as C55B3.

### 2.2. Laboratory Specimens Preparation

A laboratory steel roller compactor (EN 12697-33 [24]) was used to prepare double-layered slabs having plan dimensions of 305 × 305 mm^2^. Each layer was made of the same asphalt concrete and was compacted at a final thickness of 40 mm, considering a target air void content equal to 5.0%.

The lower layer was first compacted at 160 °C. Then, for the reinforced configurations, after slab cooling, the geocomposite was applied and the upper layer was immediately compacted at the same temperature. For the unreinforced configuration, the bituminous emulsion was spread in order to obtain 300 g/m^2^ of residual bitumen (typical dosage adopted for the tack-coat of Italian motorway pavements) and exposed to the air to allow the emulsion breaking.

Finally, for three-point bending and reflective cracking tests, two beams with 305 mm length, 100 mm width and 80 mm thickness were obtained from the central part of each slab. In the specimen beams prepared for reflective cracking tests, a 30 mm notch depth was cut at the midpoint of the lower layer, in order to simulate an existing crack. As for the three-point bending tests, part of the specimens was intended for the determination of the performance coefficient *k* (see Section 4.2.1.) and part of the specimens was intended for the determination of the *J-integral* value (see Section 4.2.2.). The first ones were prepared without any notch, whereas for the latter ones three notch depths, i.e., 10, 20 and 30 mm, were considered.

For Leutner tests, five cylindrical specimens with 100 mm diameter were cored from each slab and the compaction direction was marked.

### 2.3. Leutner Shear Test

The interlayer bond strength was measured through Leutner shear tests, performed at 20 °C. The Leutner is a pure direct shear device with no normal force applied on the interface. Following prEN 12697-48 [25], the cylindrical specimen was placed between two shear rings, spaced by a 5 mm gap. One ring moved vertically with a constant displacement rate equal to 50.8 mm/min, while the other ring was fixed and in contrast with a load cell. The interface shear displacement was applied along the compaction direction in order to simulate the field conditions. During the test, the shear force and the shear displacement were continuously measured. The interlayer shear strength (ISS) was then calculated as in Equation (1):

(1) $$\mathrm{ISS}=\frac{F_{max}}{A}=\frac{4F_{max}}{\pi D^{2}},$$

where $F_{max}$ is the maximum shear force measured by the load cell, *A* and *D* are the specimen interface area and diameter, respectively. Two replicate specimens were tested for each interface configuration.

### 2.4. Three-Point Bending Test

Three-point bending tests were performed at 20 °C in order to assess the (static) crack propagation resistance. For the determination of the performance coefficient *k* (see Section 4.2.1.) the tests were carried out on specimens without any notch, whereas for the determination of the *J-integral* value (see Section 4.2.2.) the tests were carried out on specimens with three different notch depths (10, 20 and 30 mm). In the first case, two replicate specimens were tested for each interface configuration, whereas in the second case two replicate specimens were tested for each interface configuration and notch depth. The prismatic specimens were placed on supports with a span of 240 mm and subjected to a vertical load applied at a constant rate of 50.8 mm/min. The load and the beam deflection in the middle of the specimen were measured during the test until failure by means of a load cell and a LVDT, respectively, and a digital camera was used to record the test for its entire duration.

### 2.5. Reflective Cracking Test

The “Wheel Tracker” equipment, typically used for rutting tests, was customized to perform reflective cracking tests on reinforced specimens, with the aim of assessing the ability of the geocomposites to delay the propagation of a pre-existing crack under moving loads. In particular, traffic loads were simulated through a tire wheel moving back and forward on the beam specimen. Therefore, the selected test configuration (described in details hereafter) is closer to the actual field conditions as compared to static tests.

**Figure 3:**
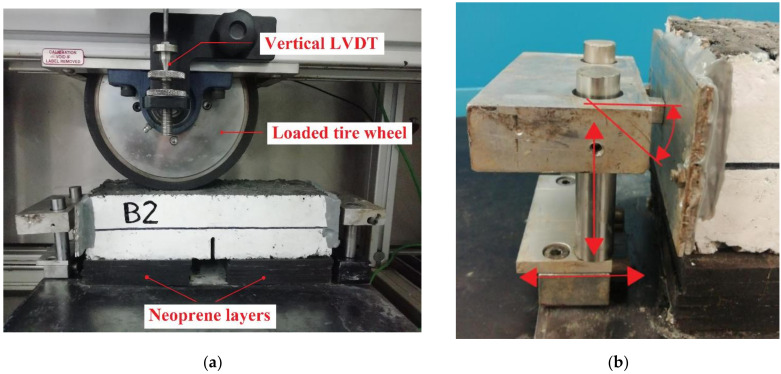
Reflective cracking test: testing setup (**a**), detail of the lateral support (**b**).

The prismatic specimen, characterized by a 30 mm notch depth in the middle of the beam, was placed on neoprene layers with a total thickness of 30 mm and a stiffness modulus of 0.3 MPa (Figure 3a). The neoprene layers, which were 35 mm apart to facilitate the crack propagation from the notch tip, simulated the bearing capacity of the subgrade soil in the field. The specimen ends were glued with an epoxy resin to jointed supports, specifically designed to let the specimen move and rotate freely, without the onset of residual forces (Figure 3b). In fact, during the test, these supports slide along two low-friction vertical guides that, in turn, slide along two low-friction horizontal guides under the load applied by the tire wheel, allowing both horizontal and vertical displacements as well as the rotation of the specimen.

The tire wheel, characterized by a diameter of 200 mm and a width of 50 mm, applies a contact force of approximately 750 N during the test and rolls back and forward on the specimen surface along a 230 mm long tracking path at a frequency of 20.7 cycles per minute. To better replicate the field conditions, the tracking path corresponds to the direction of compaction of the specimen. A vertical LVDT records the deflection along the tracking path every 0.4 mm; the deflection in the middle is considered for the analysis of the test results. In addition, a video camera placed in front of the middle section of the specimen was used to record the crack evolution during the entire test.

The tests were carried out at 30 °C and were stopped when the crack reached the upper surface of the specimen. Two specimens were tested for each reinforced configuration.

**Figure 4:**
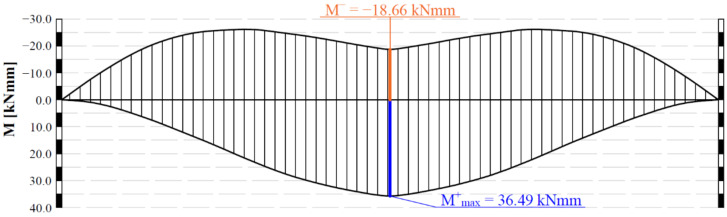
Bending moment envelope from FEM analysis.

To verify the distribution of the bending moment in the specimen during the test, a structural FEM analysis was performed. The specimen laying on the neoprene layers was modelled as a beam laying on Winkler springs. The artificial notch was considered as a reduction of the beam effective section. The bending moment envelope given by the beam own weight and the moving load is shown in Figure 4. The maximum positive moment (lower fibers in tension) is obviously in the middle and is twice the maximum negative moment (upper fibers in tension) generated in the middle when the wheel is at the extreme of the tracking path. Therefore, the selected testing conditions mainly lead to the propagation of the artificial crack towards the upper surface of the specimen, simulating the typical reflective cracking mechanism.

## 3. Field Investigation

### 3.1. Trial Section Description

**Figure 5:**
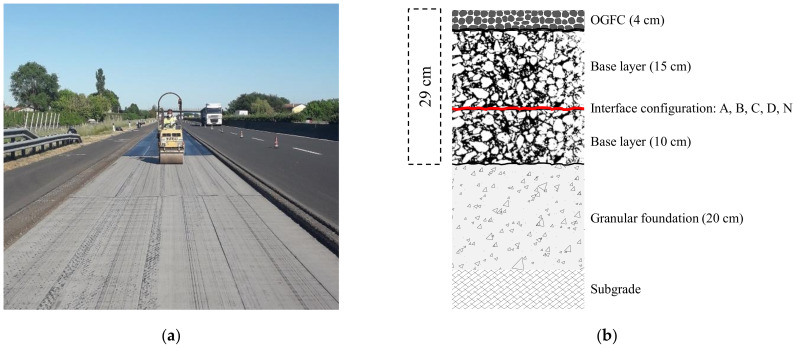
Trial section: construction phases (**a**), stratigraphy (**b**).

As a follow-up of the laboratory investigation, a full-scale trial section was constructed along the Italian A14 motorway. Specifically, a full-depth reconstruction of the slow traffic lane was carried out after milling all the existing asphalt layers (Figure 5). The new pavement consisted of an open-graded friction course (OGFC) with nominal thickness of 4 cm and two base layers with nominal thicknesses of 15 and 10 cm respectively (Figure 5b), constructed over the existing granular foundation (with nominal thickness of 20 cm).

The trial section included five consecutive test fields with a length of 100 m each: one reference test field without any reinforcement (coded as N) and four test fields with the geocomposites A, B, C and D (the same ones included in the laboratory investigation). The geocomposites were applied at the interface between the base layers, whereas a traditional bituminous emulsion was spread at the interface in the case of the unreinforced test field N.

From an operational point of view, the geocomposites (supplied by the producers in rolls 10 ÷ 15 m long and 1 m wide) were placed directly over the surface of the lower base layer (10 cm thick) and then some passages with the steel roller (Figure 5a) were carried out to further promote the adhesion with the lower layer.

As for the materials employed for the construction of the trial section, the open-graded mix used for the OGFC layer was characterized by maximum aggregate size equal to 20 mm, total bitumen content equal to 5.3% by aggregate weight and cellulose-glass fibre content equal to 0.3% by aggregate weight. Instead, the base layer mix was characterized by maximum aggregate size equal to 31.5 mm and total bitumen content equal to 4.1% by aggregate weight and contained 30% RAP. In both cases, a SBS polymer modified “hard” bitumen was used as virgin binder. The emulsion employed for the tack-coat in the unreinforced test field N was the same used in the laboratory investigation, i.e., C55B3 type according to EN 13808 [23].

It is worth pointing out that there were meaningful analogies between the field conditions and the laboratory conditions. In fact, in both cases, the geocomposites were applied at the interface between two new asphalt layers containing 25 or 30% RAP and a virgin SBS polymer modified “hard” bitumen. 

### 3.2. FWD Campaign

After the construction of the trial section, a FWD campaign was carried out to assess the stiffness of the asphalt layers in the unreinforced and reinforced test fields. The non-destructive tests were performed considering a step of about 20 m for each test field and the FWD equipment was configured with a 30 cm loading plate and nine geophones at 0, 200, 300, 400, 500, 600, 700, 800 and 1500 mm from the centre of the loading plate.

The stiffness of the layers was then determined through the back-calculation method, using ELMOD 6 software. The pavement was schematized as a three-layer elastic structure. The first layer was representative of all the asphalt layers, i.e., OGFC, 15 cm base layer and 10 cm base layer, and thus took into account also the effect of the interface configuration between the two base layers (unreinforced/reinforced with the geocomposite A, B, C or D). The second layer corresponded to the granular foundation, whereas the third layer, representative of the subgrade, was modelled as a half-space with infinite thickness. The thickness of the asphalt layers and the foundation at the measurement points was determined with the ground penetrating radar (GPR) and then considered in the back-calculation analysis.

## 4. Results and Analysis

### 4.1. Leutner Test Results

**Figure 6:**
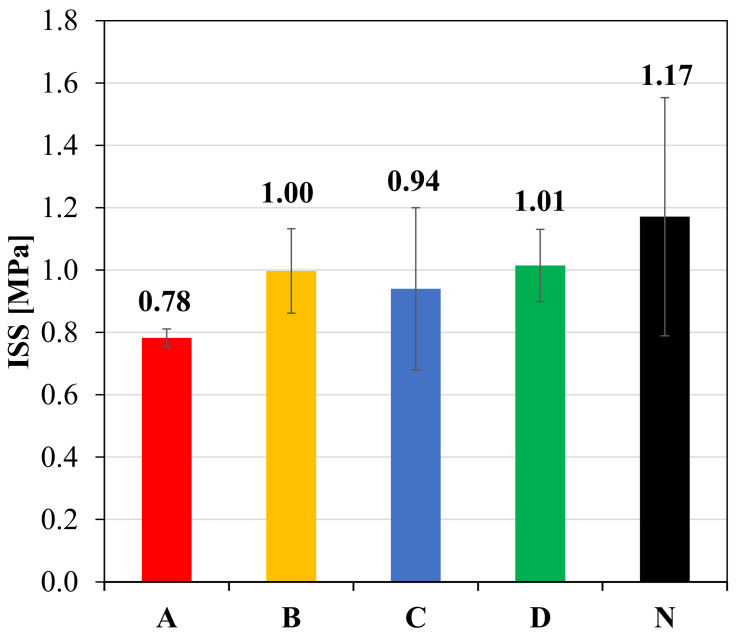
Average ISS values with error bars from Leutner tests.

The interlayer shear strength (ISS) was evaluated through Leutner tests on cored double-layered specimens. Figure 6 shows the average value of ISS and the corresponding error bars with the value of the standard deviation. 

As expected, the presence of a reinforcement decreases the adhesion between the layers as compared to the unreinforced configuration. This de-bonding effect is due to the fact that the geocomposite can be assimilated to an interlayer characterized only by a binder phase, without any solid skeleton (i.e., aggregates). Such effect can be minimized by improving the geocomposite properties, but it cannot be completely avoided, because one of the main functions of the geocomposite is indeed to provide a SAMI effect, which is possible thanks to its bituminous membrane/compound.

Comparing the performance of the geocomposites, B and D provide good shear strength at the interface, slightly higher than geocomposite C. The lowest ISS, which means the highest de-bonding effect, is associated to geocomposite A and may be due to the non-stick selvedge upper coating.

### 4.2. Three-Point Bending Test Results

#### 4.2.1. Performance Coefficient *k*

**Figure 7:**
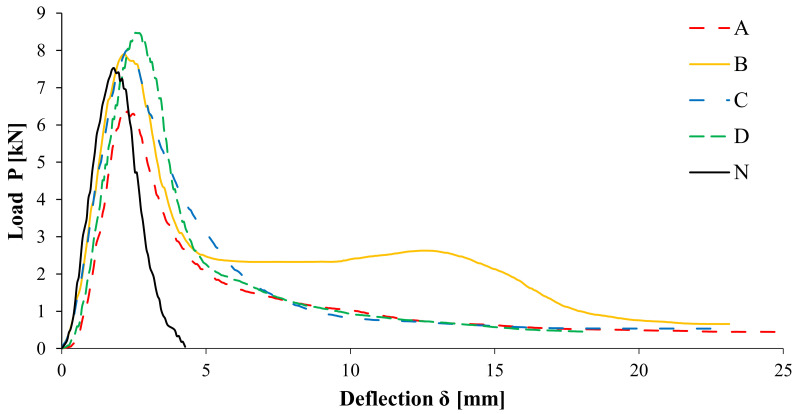
Average load-deflection curves from three-point bending tests.

The flexural behaviour of the double-layered specimens without notch was investigated by carrying out three-point bending tests, whose results are shown in Figure 7 in terms of load-deflection (*P*–*δ*) curves. The area below the curve can be divided into two contributions: the area until the flexural strength (*P_max_*) and the remaining area after *P_max_*. As confirmed also by image monitoring during the test showing no crack until *P_max_*, the first contribution can be associated to the crack initiation energy (*E_i_*), whereas the second one is an estimation of the crack propagation energy (*E_p_*). Furthermore, for double-layered specimens, *E_p_* can be considered as the sum of two contributions as follows:

(2) $$E_{p}=E_{low}+E_{up},$$

where $E_{low}$ is the energy necessary for the propagation in the lower layer and $E_{up}\;$is the energy necessary for the propagation in the upper layer [19]. In the unreinforced configuration, $E_{low}$ ($E_{low}^{UN}$) and $E_{up}$ ($E_{up}^{UN}$) are proportional to the dimensions of each layer, because the crack propagation depends only on the area of asphalt concrete involved. 

From Figure 7, it can be observed that for the unreinforced configuration (N), the load rapidly decreases after *P_max_* until complete failure and the *P*–*δ* curve is basically symmetrical. Conversely, all the reinforced configurations show a tendency to retain a residual flexural resistance also for high deflection values.

The reduction in the value of *P_max_* for the geocomposite A, unlike the other geocomposites that have maximum resistance similar to the unreinforced configuration N, may be ascribable to the de-bonding effect of the geocomposite which reduces the interlayer shear resistance, as highlighted by Leutner tests (Figure 6). It can be also noted that the geocomposites A, C and D exhibit the same post-peak behaviour with a certain dissipation. The geocomposite B, instead, shows a remarkable post-peak dissipative phase due to its multi-directional reinforcement able to absorb the applied flexural stress without loss of resistance and its great ductility (the elongation at failure of geocomposite B is about ten times higher than that of all the other geocomposites, see Table 1).

These findings confirm that the presence of the geocomposites at the interface delays the crack propagation phase in the upper layer, whereas it does not significantly affect the crack initiation phase and the propagation in the lower layer, as already observed in previous studies [19,26,27]. Hence, the propagation energy in the lower layer in the unreinforced and reinforced configurations can be considered comparable ($E_{low}^{UN}=E_{low}^{R}$), because the crack has not reached the interface yet (a small correction is necessary to take into account the actual dimensions of the specimens). As a consequence, with reference to Equation (2), the propagation energy in the upper layer in a reinforced specimen ($E_{up}^{R}$) can be calculated as the difference between the total propagation energy for the reinforced specimen ($E_{p}^{R}$) and the propagation energy in the lower layer for the unreinforced specimen ($E_{low}^{UN}$):

(3) $$E_{up}^{R}=E_{p}^{R}-E_{low}^{UN}$$

Based on the above considerations, the contribution of the geocomposite in the crack propagation phase was quantified through the performance coefficient (*k*), defined as the ratio between the crack propagation energy in the upper layer for the reinforced system ($E_{up}^{R}$) and the crack propagation energy in the upper layer for the unreinforced system ($E_{up}^{UN}$), as in Equation (4) [19]:

(4) $$k=E_{up}^{R}/E_{up}^{UN}.$$

For the reinforced configurations, a maximum deflection of 15 mm was conservatively considered. It should be noted that this choice is particularly conservative for the geocomposite B, which still exhibits a significant dissipation for deflections higher than 15 mm (Figure 7).

**Figure 8:**
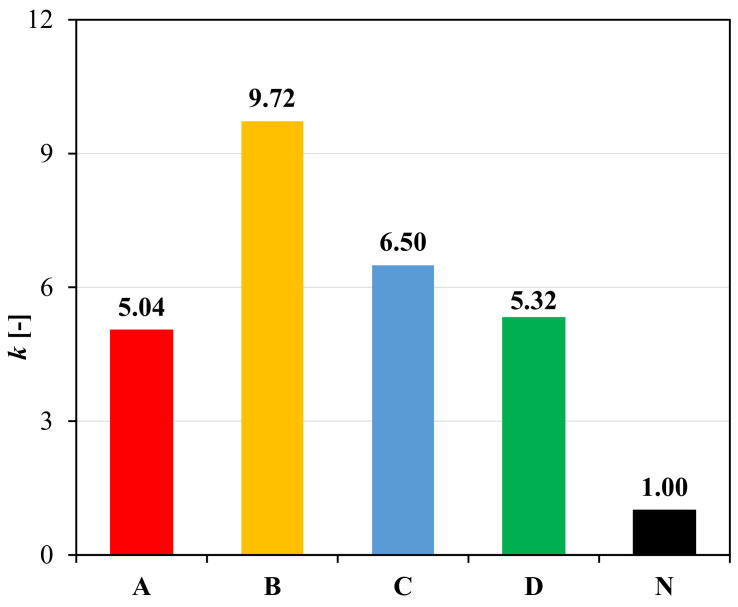
Performance coefficient *k* from three-point bending tests.

From the values of the performance coefficient *k* shown in Figure 8, it can be observed that the geocomposites increase the crack propagation energy by about five to ten times. However, B shows the best performance with a *k* value of 9.7, while the other reinforcements have a similar behaviour (as already observed from the comparison of the load-deflection curves in Figure 7) and consequently they exhibit comparable *k* values. Higher *k* value means higher capacity of the geocomposite to delay the propagation of the cracks above the reinforcement, resulting in increased service life for the pavement in terms of cracking.

#### 4.2.2. *J-Integral*

The crack propagation resistance was evaluated also by calculating the *J-integral* value, which quantifies the strain energy release rate, using the following equation:

(5) $$J-integral=-\frac{1}{B}\left(\frac{d\;U_{15mm}}{da}\right),$$

where *B*, *a* and $U_{15mm}$ are the specimen width, the notch depth, and the total strain energy calculated considering the area under the load-deflection curve up to a deflection of 15 mm (the same maximum deflection considered to calculate the performance coefficient *k*). Therefore, $\frac{d\;U_{15mm}}{da}$ represents the rate of change of the fracture energy as a function of the notch depth. Higher *J-integral* values indicate better fracture resistance.

**Figure 9:**
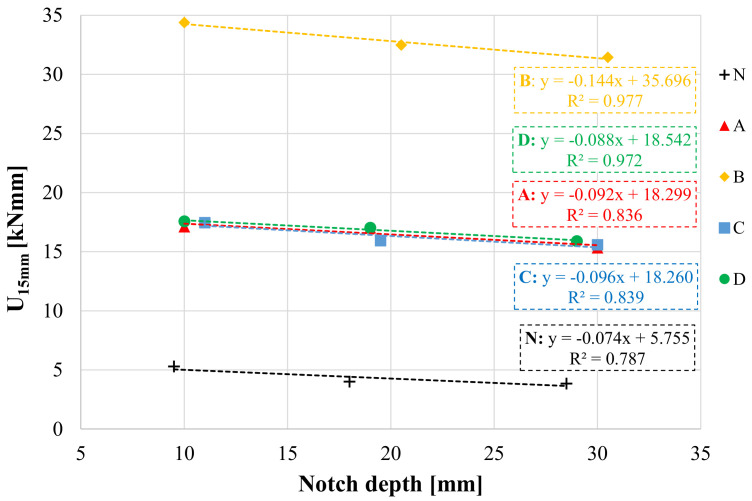
Strain energy *U* calculated until a deflection of 15 mm for different notch depths and different interface configurations.

**Figure 10:**
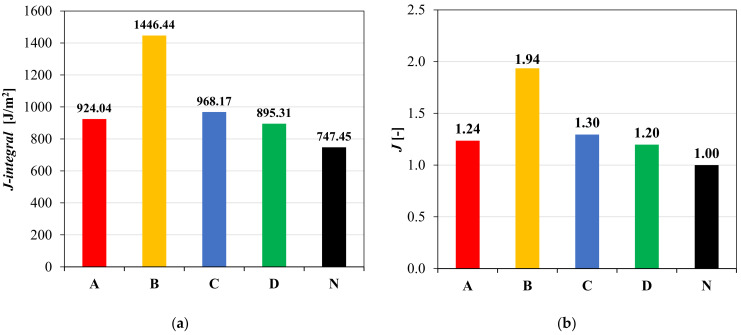
*J-integral* (**a**) and *J* ratio (**b**) values for different interface configurations from three-point bending tests.

Figure 9 presents the results obtained from the three-point bending tests carried out on notched specimens. The strain energy, calculated as average value of two replicates, decreases with an increase in the notch depth. The slope of the linear fitting was then divided by the specimen width to calculate the *J-integral* according to Equation (5). The results obtained are summarized in Figure 10a. 

In addition, in order to compare these results with the results obtained in terms of performance coefficient *k*, the *J* ratio was defined as in Equation (6):

(6) $$J=J\text{-}integral^{R}/J\text{-}integral^{N},$$

where $J\text{-}integral^{R}$ and $J\text{-}integral^{N}\;$are the *J-integral* values for the reinforced and unreinforced system, respectively. The results in terms of *J* ratio are shown in Figure 10b.

The results presented in Figure 10a,b indicate that all the reinforced configurations have better cracking performance than the unreinforced one. The high cracking resistance of the specimens with geocomposite B may be attributed to its great tensile elongation at failure, which is approximately ten times higher than that of the other geocomposites (Table 1). As already observed from the load-deflection curves shown in Figure 7, the geocomposites A, C and D have, in general, a similar behaviour due to the similar characteristics of their reinforcements. However, based on the *k* parameter and *J* ratio values (Figure 8 and Figure 10b), the geocomposite C shows slightly better performance than A and D.

**Figure 11:**
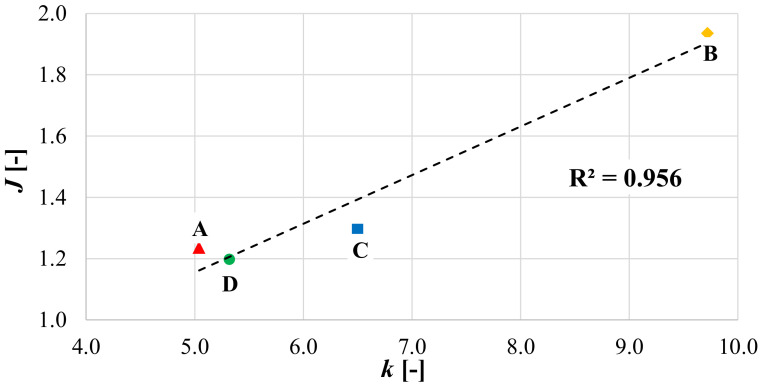
Correlation between the performance coefficient *k* and the *J* ratio.

An attempt was made to find a relationship between the performance coefficient *k* and the *J* ratio. Figure 11 shows that there seems to be a linear relationship between *k* and *J*, indicating that both parameters could characterize the ability of the geocomposites to delay the crack propagation. However, the *k* values are between 5 (geocomposite A) and 9.7 (geocomposite B), while the *J* values are included between 1.2 (geocomposite D) and 1.9 (geocomposite B). Therefore, it seems that the *J* parameter does not allow to fully catch and quantify the differences between the reinforced systems and the unreinforced one in terms of energy required for the crack propagation.

In order to explain this finding, the meaning of the *J-integral* should be considered. The *J-integral* method was introduced independently by Cherepanov [28] and Rice [29], and it is applicable in materials for which the deformation is mostly inelastic. For these materials, a significant part of the strain energy is dissipated within the plastic zone and the rest leads to the propagation of the crack. Moreover, a fundamental hypothesis of the *J-integral* method is that the body should be homogeneous [29]. However, it must be underlined that such hypothesis is not valid in the case of reinforced double-layered asphalt specimens due to the presence of the geocomposite, which represents a singularity.

Therefore, for the above-mentioned reasons, the performance coefficient *k* seems more reliable than the *J* parameter to quantify the contribution of the geocomposite in the crack propagation phase and can be used in pavement design to amplify the crack propagation resistance of the asphalt layers above the geocomposite. In addition, varying in a wider range, *k* allows to better distinguish the properties of different geocomposites.

### 4.3. Reflective Cracking Test Results

**Figure 12:**
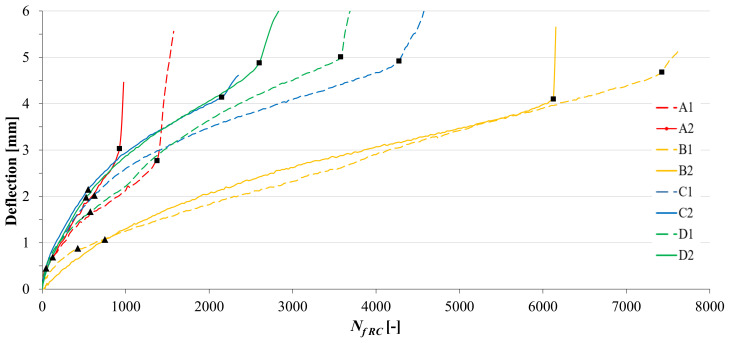
Results of the reflective cracking test.

The reflective cracking tests were carried out in order to investigate the crack propagation resistance of the double-layered reinforced specimens with a pre-existing crack under cyclic loading. The results (Figure 12) are represented in terms of vertical deflection in the middle of the specimen vs. applied load cycles.

**Figure 13:**
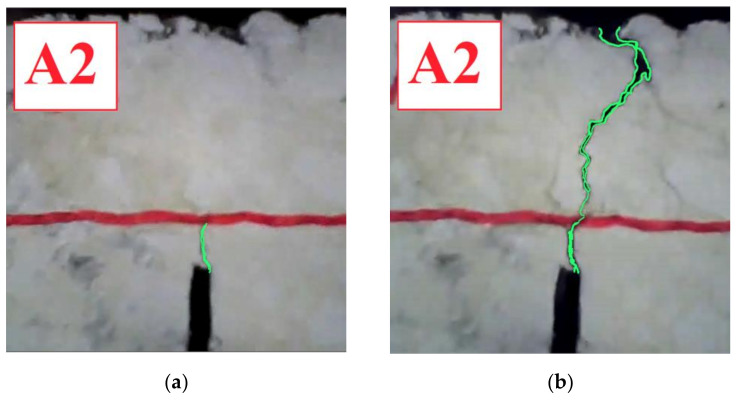
Crack evolution in a specimen with geocomposite A: beginning of the second (**a**) and third (**b**) stages.

The typical evolutive curve is characterized by three stages, identified by a fast change of the slope as follows:

- *First stage*: the specimen is intact. The final point of the first stage is indicated as a black triangle on the curves in Figure 12.
- *Second stage*: a crack originates from the notch and quickly reaches the geocomposite. From now on, the geocomposite plays a major role in delaying the crack propagation in the upper layer. This second phase is almost linear and the slope represents the crack propagation rate. The final point of the second stage, indicated as a black square on the curves in Figure 12, was considered as the specimen failure, at which the number of cycles to failure *N_f RC_* was computed. The initial and final points of the second stage were identified thanks also to the analysis of the recording made during the tests, as shown in Figure 13 (where the front surface of the specimen is painted in white, the interface is marked in red and the cracks are highlighted in green). The crack propagation rate and the *N_f RC_* values obtained for all reinforced systems are summarized in Table 2.
- *Third stage*: the crack is clearly evident on the upper surface of the specimen and high deflections are registered.

As it can be seen from Figure 12, the systems with the geocomposites A, C and D have a similar behaviour in the first stage, whereas geocomposite B has the best deformation resistance in this stage. However, for all reinforced configurations, the first stage is about 500 cycles long, confirming that the geocomposites do not have a significant effect on delaying the crack initiation.

The second stage is the most important to evaluate the performance of the double-layered systems. A single crack quickly propagated from the notch to the upper specimen surface for the geocomposite A, leading to a brittle failure which resulted in the lowest number of cycles to failure (Table 2). This may be attributed to a high slippage at the interface, which was not able to ensure adequate bonding between the asphalt layers, as suggested by Leutner tests results (Figure 6). As a consequence of the de-bonding effect, the upper layer was almost completely separated from the lower layer, leading to a significant reduction of the moment of inertia of the system (the height of the resisting section in the middle was 40 mm instead of 50 mm). It is worth noting that also the three-point bending test results (Figure 7) indicated that the systems with the geocomposite A had a lower flexural strength (*P_max_*), most likely due to the de-bonding effect of the reinforcement ascribable to its different upper coating made of a non-stick selvedge (Figure 2).

**Figure 14:**
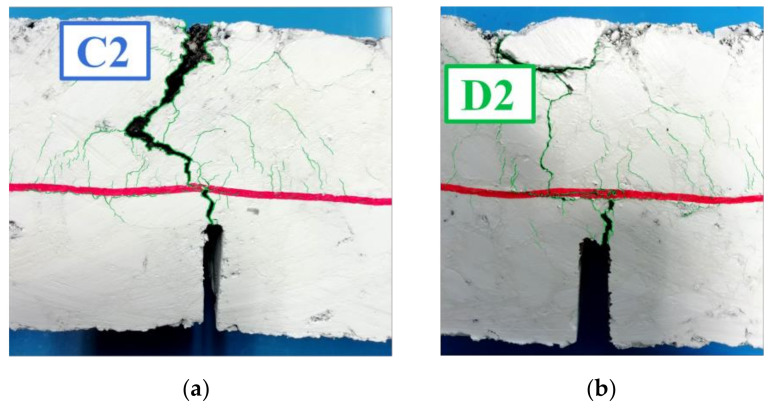
Failure mechanism observed for the specimens with geocomposite C (**a**) and D (**b**).

Less slippage occurred at the interface for the geocomposites C and D. These reinforcements had a similar behaviour, leading to sub-horizontal cracks at the interface without loss of resistance, thus acting as a SAMI and increasing the number of cycles to failure by delaying the appearance of the crack on the upper specimen surface. In fact, the upper asphalt layer was characterized by several interconnected microcracks which initiated at different points of the geocomposite surface, as can be seen in Figure 14. Based on literature, this mechanism can be defined as a controlled de-bonding [1]. Considering the comparison of the parameters in Table 2, the performance of the geocomposites C and D is intermediate between A and B.

On the contrary, no slippage occurred at the interface of the systems with the geocomposite B. Its multi-directional fiberglass (different from the glass-grids of the other geocomposites) had a strong bonding effect, which made the specimen act as a whole, as demonstrated by the lowest value of the crack propagation rate (Table 2). The geocomposite was able to provide a good bonding (Figure 6), promoting stress-relieving and ensuring a number of cycles to failure twice as high as C or D systems. As already discussed in Section 4.2.1 and Section 4.2.2, the superior performance of geocomposite B is ascribable to its high ductility, in particular to its tensile elongation at failure that is ten times higher than all the other geocomposites (Table 1).

**Figure 15:**
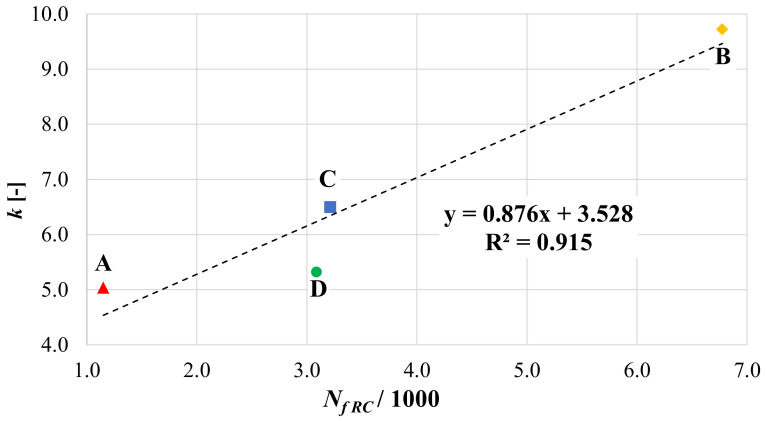
Correlation between the performance coefficient *k* and the number of cycles to failure *N_f RC_* from the reflective cracking test.

A possible correlation was then sought between the results of the reflective cracking tests and the results of the three-point bending tests. A linear correlation was found between *N_f RC_* and *k*, as shown in Figure 15. This correlation indicates that both test methods (and parameters) are able to characterize the flexural behavior of reinforced systems with delayed bottom-up crack propagation. This correlation (which needs to be corroborated by additional tests) is particularly useful because it allows to predict the performance under cyclic loads from simple static tests, which require considerably shorter times in terms of specimen preparation and testing time.

### 4.4. FWD Test Results

**Figure 16:**
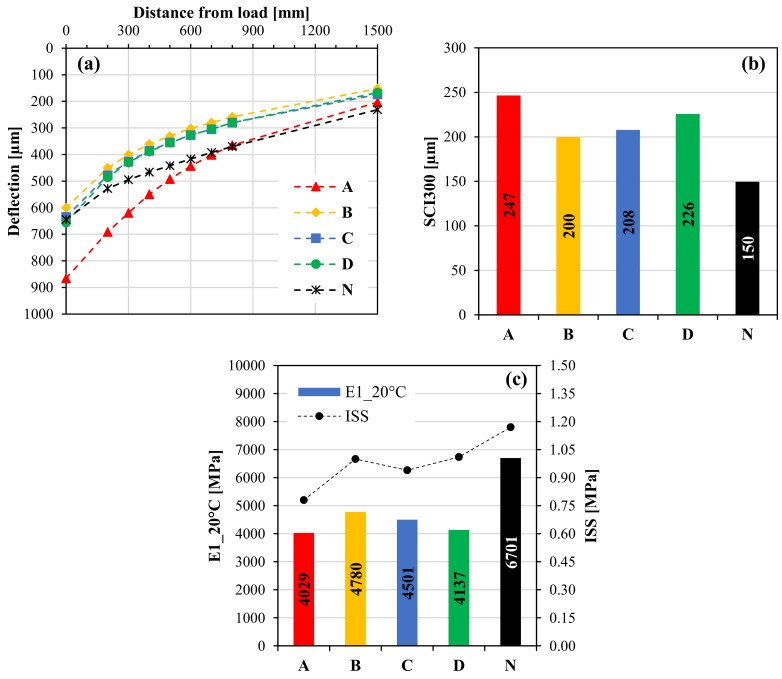
Results of the FWD tests: representative basins (**a**), SCI300 values (**b**), stiffness moduli of the asphalt layers, compared with the average ISS values (**c**).

Figure 16 includes the results obtained from the FWD tests and the related back-calculation analysis. Specifically, Figure 16a shows the representative basin for each test field, i.e., the measured basin closest to the average one. Figure 16b shows the values of the surface curvature index SCI300, calculated as the difference between the deflection under the loading plate and the deflection at 300 mm from the loading plate. This index is indicative of the bearing capacity of the upper layers of the pavement (i.e., asphalt layers), and lower values of SCI300 imply greater bearing capacity. Figure 16c instead shows the values of E1_20 °C, which is the stiffness modulus of the asphalt layers obtained from the representative basin through the back-calculation and referred to a temperature of 20 °C (for comparison purposes). It should be recalled that the meaning of this modulus can be associated to an “equivalent modulus” because it is representative of an overall response given by the 4 cm OGFC, the 15 cm base layer and the 10 cm base layer and takes into account also the bonding conditions at the interface between the two base layers (unreinforced/reinforced with the geocomposite A, B, C or D). Moreover, for a better interpretation of the results, the average interlayer shear strength (ISS) values obtained from Leutner tests (Figure 6) are shown on the secondary axis in Figure 16c.

From Figure 16a, it should be noted that the deflection values measured at 1500 mm from the loading plate (which mainly depend on the properties of the lower layers, i.e., foundation and subgrade) are generally comparable for all test fields, indicating that the different deflections measured close to the loading plate are mostly attributable to the properties of the asphalt layers. In this regard, it can be observed that the test field A clearly exhibits the highest deflections, whereas the other three reinforced test fields (B, C and D) exhibit similar deflections, much lower than those of the test field A.

As compared to the test fields B, C and D, the unreinforced pavement (N) shows a similar deflection under the loading plate and greater deflections distant from the loading plate. However, it should be emphasized that, as the distance from the load increases, the reduction in the deflection for the unreinforced test field is much lower than that observed for all reinforced pavements. This is confirmed also by the SCI300 values shown in Figure 16b, which suggest that the bearing capacity of the asphalt layers is significantly greater for the unreinforced pavement (N) with respect to all the test field with the geocomposites. Moreover, Figure 16b indicates also that the lowest bearing capacity of the asphalt layers emerges for the test field A.

Consequently, from Figure 16c, it can be observed that the unreinforced pavement (N) exhibits the highest “equivalent” stiffness modulus, which is significantly greater than that of the reinforced test fields. This difference is ascribable to the de-bonding effect of all geocomposites, which is demonstrated by the ISS reduction with respect to the unreinforced system. This finding confirms that the reinforcement effect provided by the geocomposite is not linked to an increase in the pavement stiffness but rather to the limitation of the crack propagation phase, as already demonstrated by previous studies [30]. It is worth pointing out that the stiffness reduction caused by the presence of the geocomposite is not necessarily a negative aspect, but it should be properly taken into account (for instance, it might imply an increase in the strain/stress level at the bottom of the asphalt layers).

Instead, the differences between the reinforced test fields in terms of E1_20 °C are less evident from Figure 16c. Nevertheless, it can be noted that the test field A shows the lowest stiffness modulus among the test fields with the geocomposites, along with the lowest ISS value. The stiffness of the other test fields is somewhat higher (especially for B and C), in line with higher ISS values. Therefore, reinforcements ensuring good adhesion between the asphalt layers should be used to minimize the stiffness reduction caused by the presence of the reinforcement.

## 5. Conclusions

The objective of this study was to evaluate the influence of four geocomposites with different physical and mechanical properties on the crack propagation and interlayer bonding of asphalt pavements. First, a laboratory investigation was carried out on double-layered asphalt specimens, which were subjected to three-point bending, reflective cracking and Leutner tests. Then, a trial section was constructed along an Italian motorway and a FWD campaign was carried out.

The laboratory investigation highlighted that the main contribution of the geocomposites consisted in increasing the crack propagation energy in the layer above the reinforcement (from five to ten times with respect to the unreinforced system), indicating that geocomposites can significantly extend the service life of the pavement by delaying bottom-up and reflective cracking. This aspect can be properly considered in pavement design by amplifying the crack propagation resistance of the asphalt layers above the geocomposite through the proposed performance coefficient *k*. On the other hand, all the geocomposites worsened the interlayer bonding between the asphalt layers as compared to the unreinforced system (de-bonding effect).

The field investigation indicated that all the geocomposites decreased the stiffness of the asphalt layers with respect to the unreinforced pavement as a consequence of the de-bonding effect, thus corroborating the laboratory results.

The study also showed that the geocomposite properties play a major role on the overall performance of the reinforced pavement. A high energy dissipation capability and an upper coating ensuring good adhesion between the asphalt layers are recommended to maximize the delay of the crack propagation and minimize the de-bonding effect.

The constant monitoring of the existing trial section in the future will provide valuable data on the long-term field performance of reinforced pavements subjected to actual motorway traffic.

## Figures and Tables

**Table 1:** Mechanical properties of the geocomposites.

	A	B	C	D
Nominal tensile strength L/T (kN/m)	40/40	35/35	40/44	40/40
Tensile elongation at failure L/T (%)	4/4	30/30	3/3.5	4/4

**Table 2:** Synthetic parameters obtained from reflective cracking tests for different interface configurations.

	A	B	C	D
Crack propagation rate (mm/1000 cycles)	2.19	0.52	0.94	1.18
Number of cycles to failure, *N_f RC_*	1150	6775	3213	3088

## Data Availability

The data presented in this paper are available upon request from the corresponding author.
